# Supplementary figures and images for: Cerebrospinal Fluid EV Concentration and Size Are Altered in Alzheimer’s Disease and Dementia with Lewy Bodies
Source: Cells. 2022 Jan 28;11(3):462. doi: 10.3390/cells11030462 (PMC8834088; doi:10.3390/cells11030462)

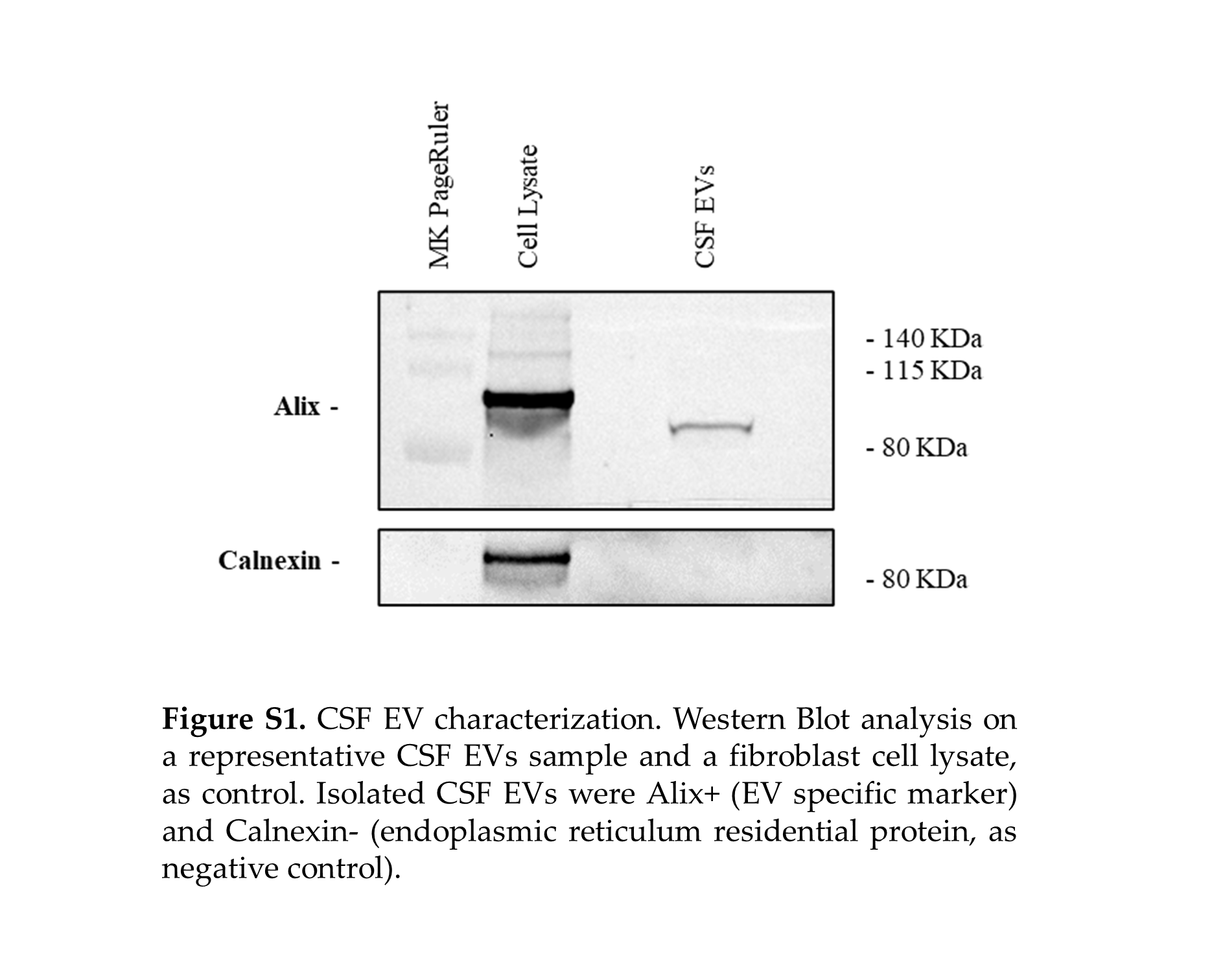

Supplement: Supplementary file 1 [file cells-11-00462-s001.zip › Figure S1.tif]

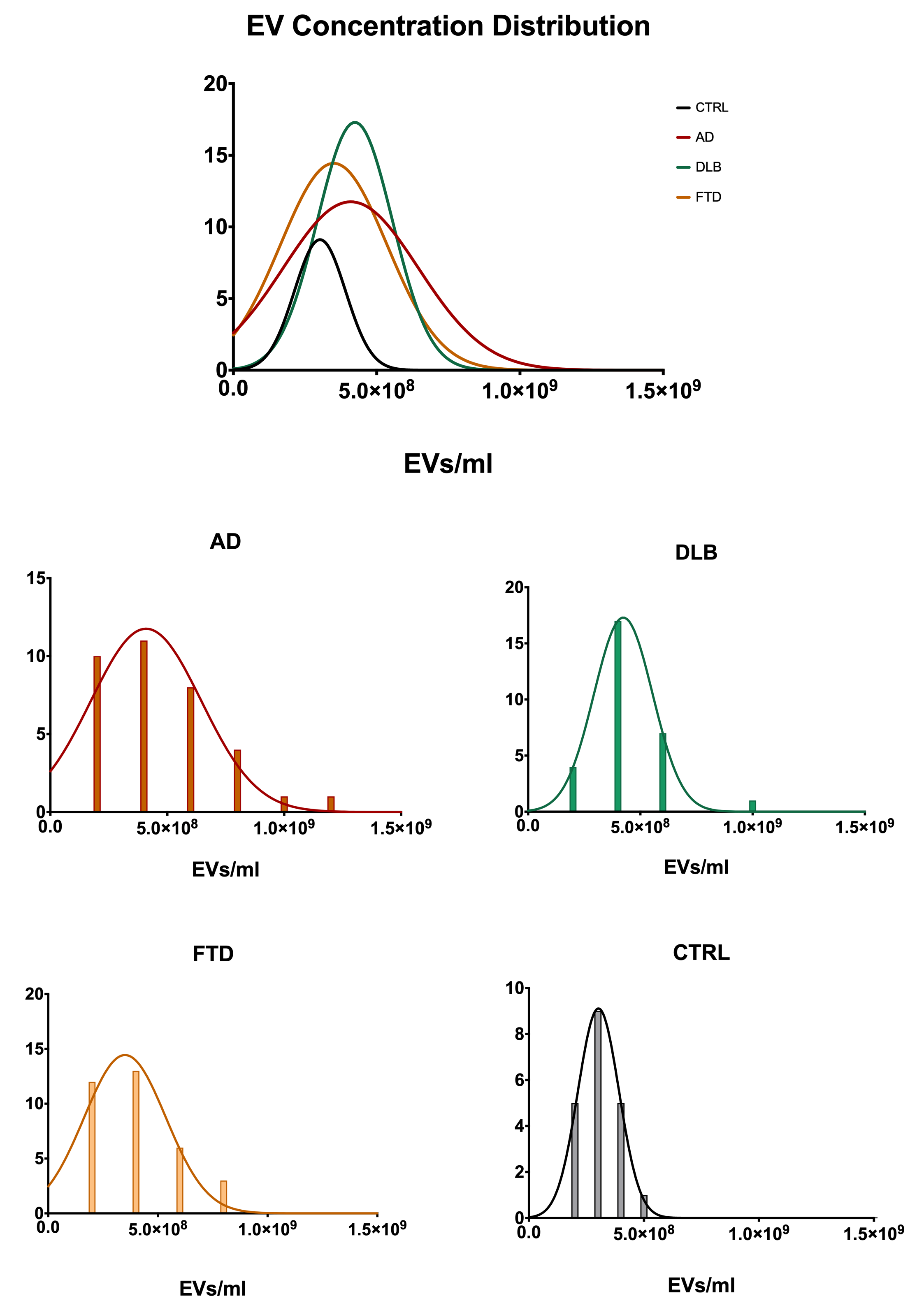

Supplement: Supplementary file 1 [file cells-11-00462-s001.zip › Figure S2.tiff]

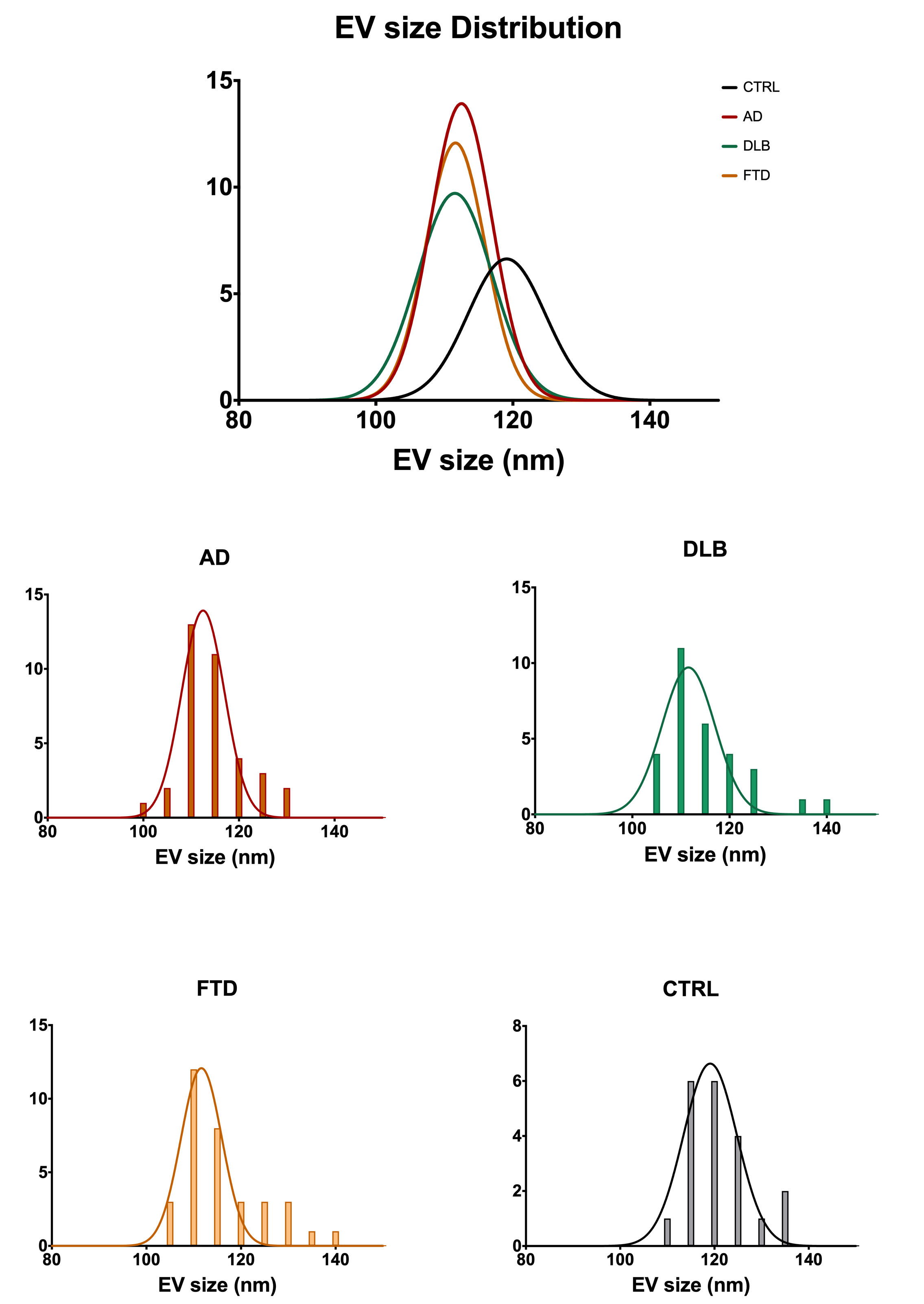

Supplement: Supplementary file 1 [file cells-11-00462-s001.zip › Figure S3.tiff]

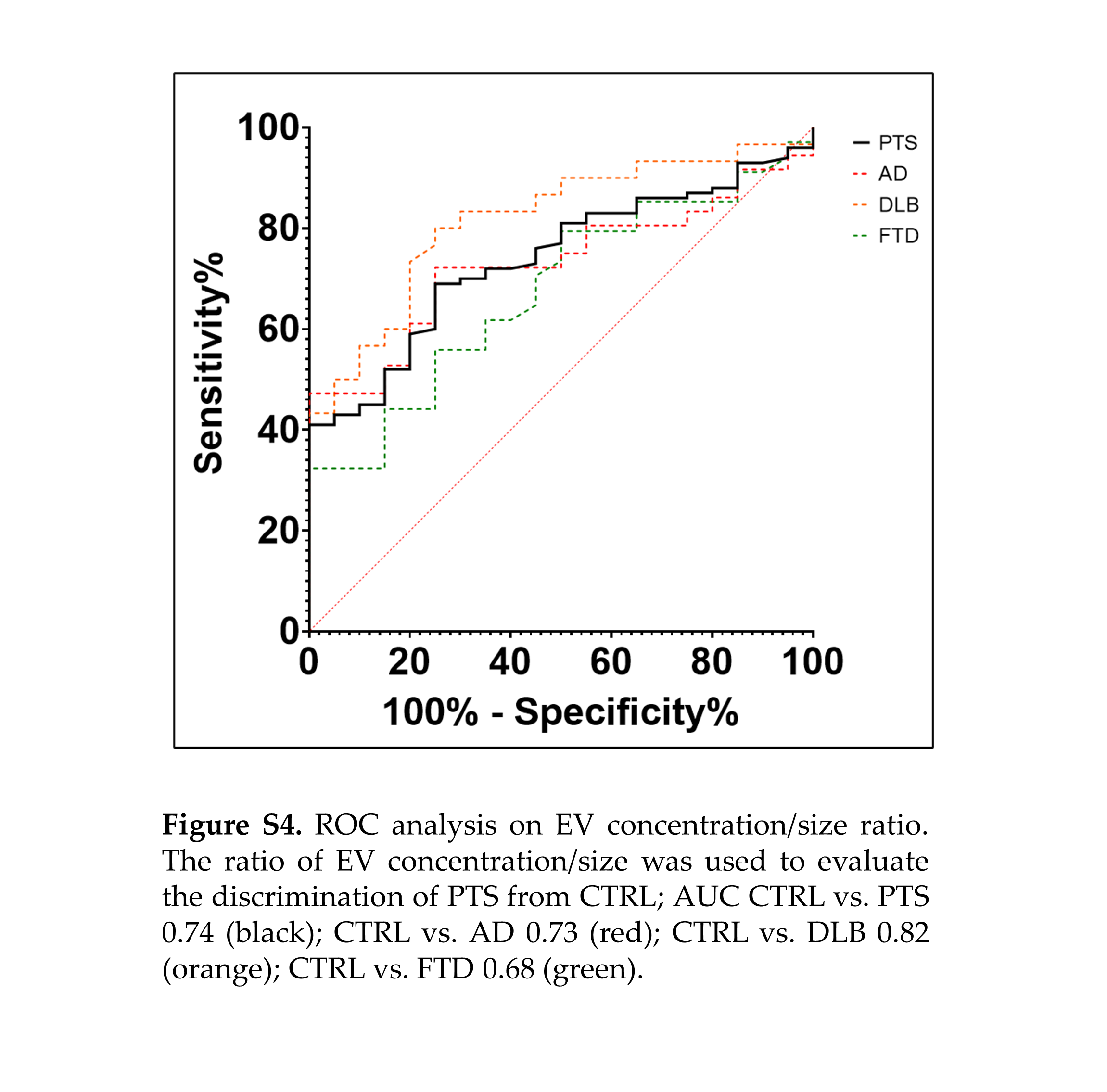

Supplement: Supplementary file 1 [file cells-11-00462-s001.zip › Figure S4.tif]
